# Travels in Gene Space

**DOI:** 10.3201/eid1504.080823

**Published:** 2009-04

**Authors:** Julian W. Tang

**Affiliations:** National University Hospital, Singapore

**Keywords:** gene sequences, another dimension

The Captain peers over the bow of his computer ship,
Floating down the river of evolution-time,
In the landscape of gene-space.
Gene sequences running parallel on either side,
Spreading, evolving, as far as the eye can see.
Ahead, the future is unclear, sequences shrouded in mist.
But this is no surprise.
Prediction is a risky business.
Looking behind, some sequences shine brightly,
Known samples, from known times.
Others are blurred in the mists of time past.
The Captain taps his keyboard.
Some of the mist dissipates,
And some sequences become visible
In the light of inference.
An epidemic history revealed perhaps?
The Captain sighs and taps his keyboard again.
His environment changes.
He is now surrounded by hills and valleys,
With moving figures all around him.
Markov Chain Monte Carlo robots,
Walking, climbing, up and down.
Some, aimlessly wandering in circles,
In the undulating landscape of tree-space.
He taps his keyboard once more.
The MCMC robots change direction,
Seemingly, becoming more purposeful.
The Captain allows himself a smile.
Suddenly, he feels a tug on his leg.
Looking down, he sees the cherubic face
Of his young son, looking up at him.
“Can we play football, daddy?”

